# Impact of selected baking and vacuum cooling parameters on the quality of toast bread

**DOI:** 10.1007/s13197-020-04945-x

**Published:** 2021-02-08

**Authors:** Mathias Kinner, Ramona Rüegg, Claudia A. Weber, Jürg Buchli, Ludwig Durrer, Nadina Müller

**Affiliations:** 1 Zurich University of Applied Sciences, Grüental Postfach, 8820 Wädenswil, Switzerland; 2 Durrer Spezialmaschinen AG, Calendariaweg 2, 6405 Immensee, Switzerland

**Keywords:** vacuum cooling, Toast bread, Oven temperature, Core temperature

## Abstract

Vacuum cooling of baked goods can deliver many advantages in terms of product quality and productivity, such as higher volumes and shorter cooling times. However, the associated high costs and the need to adjust baking protocols are of relevance and more information is needed. This paper examines the influence of two main baking protocol parameters on the quality of toast bread, i.e. oven temperature and baking time reduction. Resulting toast bread characteristics including specific bread volume, concavity, browning index, crust and crumb hardness and a_w_-value were analysed as well as process-dependent core temperature and water loss. In order to compensate for water loss during vacuum cooling and still achieve optimal toast bread quality, a final bread core temperature of 98 °C at the end of baking gave best results, regardless of oven temperature. It was further shown that cooling time can be reduced by a factor of 10 if the baking protocol is optimally adjusted, hinting at a huge potential to increase productivity for industrial applications. In summary, it can be stated that vacuum cooling requires a tailored reduction in baking time in order to compensate for water loss from vacuum cooling while retaining sufficient structural cohesion to resist deformation of the bread.

## Introduction

Vacuum cooling is based on the thermodynamic principle of adiabatic cooling, in which water from the product is evaporated by lowering atmospheric pressure, which forces water to phase transition using the latent heat of the product (McDonald and Sun 2000; Zheng and Sun 2004). As a consequence, processing equipment used for vacuum cooling is built and works differently (Sun and Zheng 2006) to common cooling systems, which are based on the principle of heat exchange. Various effects of the application of vacuum cooling have been described for several food categories such as meat (Sun and Wang 2000; Mc Donald and Sun 2001), fruits (Li et al. 2017) and vegetables (Rodrigues et al. 2012, 2013). The most dominant advantage of vacuum cooling is its markedly reduced process time compared to conventional cooling, unfortunately accompanied by the fact that vacuum cooling is a highly product specific process (Sun and Zheng 2006). To provide better understanding of vacuum cooling and the respective heat and mass transfers, mathematical and simulation models have been developed for liquid food, vegetables and cooked meat, respectively, and effects of specific process parameters estimated (Zhu et al. 2019).

In the context of baked goods, vacuum cooling has been applied to tailor product quality: Primo-Martin et al. (2008) have shown that vacuum cooling can positively affect crispiness of the crust. However, a higher water loss was observed and therefore a reduced baking time suggested to compensate water loss. A negative effect on staling of bread has been found where no reduction of baking time was implemented (Le-Bail et al. 2011). Novotni et al. (2017) investigated the influence of barley sourdough and vacuum cooling on shelf life quality of partially baked bread. It was shown that additional vacuum cooling improved bread shape, porosity and reduced sour taste, crust colouring and crumbliness. Albeit the baking time was not reduced for vacuum cooled partially baked breads, it was reduced for the final baking step.

In summary, a shortening of baking time leading to changes in core temperature was not subject to research in combination with vacuum cooling yet, neither was the variation of oven temperature in order to maintain a product specific crust colour. Both shortening baking time and raising of oven temperature are options to compensate for the effect of vacuum cooling. However, both of these interventions influence the structure of the baked goods since there is a clear correlation between core temperature and various reactions such as protein denaturation, starch gelatinisation and formation of aroma compounds (Besbes et al. 2013; Roth 2007; Ben Aissa et al. 2010). Because toast bread has a very thin and soft crust and a large number of very small pores, it is crucial to optimise baking time in order to preserve the desired qualities of the final loaf.

This work aimed to explain core interactions between baking parameters, vacuum cooling and quality attributes, whereby toast bread served as a globally well-known model for baked goods.

## Material and methods

### Preparation of standard toast bread

The recipe and process to produce the toast bread are based on industrial know-how as well as standard developed in pretrials which allow the adaptation of industrial protocols to the pilot scale infrastructure. To prepare the dough, 2.7 kg wheat flour (type 550 IPS, Willi Grüninger AG, Switzerland; 3 °C), 1.215 kg tap water (3 °C), 0.5 kg ice flakes (Koch Kälte AG, Switzerland), 0.11 kg standard baker yeast (Hefe Schweiz AG, Switzerland; 5 °C), 0.055 kg salt (“Jura Sel”, Schweizer Salinen AG, Switzerland), 0.055 kg rapeseed oil (Holl-Rapsöl, Florin AG, Switzerland; 5 °C) and 0.027 kg baking improver (Premix Soft’r Alpaga, Puratos AG, Switzerland) were mixed and kneaded for 300 s at 20 s^−1^, 240 s at 40 s^−1^, 300 s at 45 s^−1^ and 120 s at 50 s^−1^ (Spiralkneter DIOSNA Dierks & Söhne GmbH, Germany). Total amount of dough was selected in pretrials to achieve optimal kneading results and the target dough temperature was 24.5 ± 1 °C. After 10 min rest in the kneader, 2.812 kg of the dough was weighed out for further processing, followed again by a 10 min rest period at room temperature. For dividing the dough into 0.093 kg portions a Quadro-King 2000 (Huwiler technics, Switzerland) at settings of room height 8 and intensity 1 was used in order to gain optimised final pore structure in toast bread (adapted to meet industrial settings). Eight dough portions thereof were transferred into each of a total of six greased baking tins with lids (Pitec AG, Schweiz, l: 300 mm, w: 90 mm, h: 90 mm; grease: Trennwachs 30, Dübör FRANCE S.A.S., France). Fermentation took place for 40 min at 30 °C and 75–80% RH in a climate-controlled cabinet (KOFI LINE, Kolb Kälte AG, Switzerland).

Baking and cooling procedures were aligned with best practice from industry and have been tested in various pretrials. The bread was baked for 20 min at 230 °C (steam emerging from 200 mL water was added at the beginning; steam draft closed), followed by 15 min at 220 °C (steam draft closed) and finally 15 min at 200 °C with the steam draft open (MIWE condo, Germany). After baking, the toast bread loaves were removed from their tins, placed on a flat baking tray and transferred to a tempering chamber (MIWE R 449, Germany) at 65% RH and 20 °C. The core temperature was measured (testo 925, Testo AG, Germany) and cooling was stopped as soon as a core temperature of 35 °C had been reached. Standard toast bread was produced in duplicate on different days resulting in 12 baked loaves in total.

### Preparation of vacuum cooled toast bread

Dough was prepared in triplicate using the same procedure as for the standard toast loaves resulting in 18 baked loaves in total. The baking temperatures were kept constant at either 200, 220 or 240 °C (MIWE condo, Germany). Steam emerging from 200 mL water was added at the beginning, and from 100 mL water after 17 min. The baking time was defined as the time required to achieve a corresponding core temperature of 90, 95 or 98 °C for baking temperatures of 200, 220 or 240 °C, respectively. Selection was made based on pilot settings adapted to industrial standard, hence integrating lower and upper limits of resulting final toast bread quality. The core temperature was measured (ALMEMO 2690-8A, Ahlborn Almemo Mess- und Regelungstechnik GmbH, Germany) for one toast bread loaf (l: 150 mm, w: 45 mm, h: 45 mm) per batch. The core temperature followed a sigmoidal curve during baking at all three oven temperatures as also shown by Bredariol et al. (2019). Average baking times at 200 °C oven temperature were 24.0, 26.8 and 28.8 min for 90, 95 and 98 °C bread core temperature, respectively. At an oven temperature of 220 °C average baking time to reach 90, 95 and 98 °C bread core temperature was 21.2, 22.5 and 26.8 min, respectively. Baking times of 18.8, 21.3 and 28.3 min were needed at the oven temperature of 240 °C to reach bread core temperatures of 90, 95 and 98 °C.

**Fig. 1:**
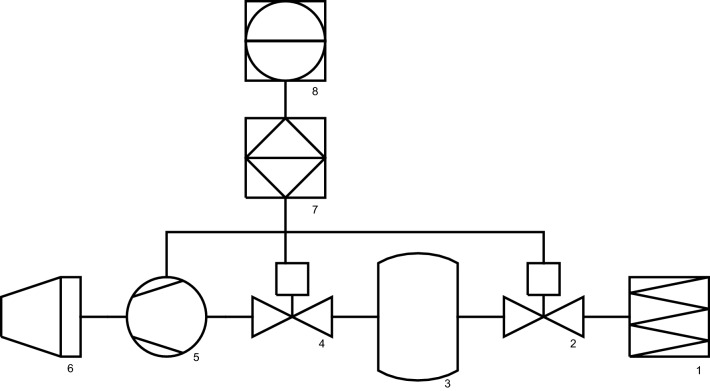
Scheme of vacuum chamber: *1* particle filter, *2* controllable valve, *3* vacuum vessel, *4* pressure regulating unit, *5* vacuum pump, *6* exhaust with silencer, *7* control system, *8* control device

After baking and weighing, the baking tins with the toast bread loaves were placed in the pilot plant vacuum chamber (prototype in pilot plant scale; scheme according to Fig. 1). The cooling process was initiated 2 min after baking. Based on industrial experience and know-how adapted in pretrials to the properties of the pilot plant vacuum chamber, the cooling protocol was defined with following steps: 100 s to reach 100 mbar, 30 s to 80 mbar, 10 s to 75 mbar, 30 s to 70 mbar, 20 s to 65 mbar and 120 s to 60 mbar. The final pressure of 60 mbar corresponds to a core temperature of 35 °C (Sun and Zheng, 2006).

### Weight loss over process time

To determine the weight loss during the process, five toast breads were weighed before and after baking, as well as after cooling/vacuum cooling using a scale (Spider SW, Mettler Toledo GmbH, Germany). The proportional weight loss was calculated with Eq. (1).

1 $$\textit{weight}{\ }\textit{loss}{ }\left[ {{\% }} \right] = 100 - { }\frac{{100 \ {*} \ \textit{weight}\;\textit{after}\;\textit{process}\,\textit{step}{ }\left[ g \right]}}{{\textit{weight}\;\textit{before}\;\textit{process}\,\textit{step}{ }\left[ g \right]}}$$

### Analysis of toast bread quality

Immediately after cooling, the toast bread loaves were analysed. The order of analysis was the same for all samples, i.e. (1) specific volume, (2) browning index, (3) crust hardness, (4) Concavity, (5) crumb texture and (6) water activity (a_w_-value).

#### Specific volume

To determine the specific volume, the weight was measured using a scale (Spider SW, Mettler Toledo GmbH, Germany) and the volume was measured using a digital volume measuring instrument (Bread Vol Scan, Pregesbauer, Austria) of five toast bread loaves each. The specific volume $\vartheta$ was calculated according to Eq. (2).

2 $$\textit{specific}\ \textit{volume}\ (\vartheta) \left[ \frac{l}{kg} \right] = \frac{\textit{volume} \left[ l \right]*100}{{\textit{weight}\ \textit{of}\ \textit{toast}\ \textit{bread} \left[ {kg} \right]}}$$

#### Browning index

To investigate the browning index, the colour at three equidistant points on the top of three toast bread loaves was analysed using L- a*-b* system in a colorimeter (CR-400, Konica Minolta, INC, Japan). The data was used to calculate the browning index (BI) with Eq. (3) according to Palou et al. (1999).

3 $$browning\, index\,\left( {BI} \right) \left[ - \right] = \frac{{100*\left( {\frac{{\left( {a + 1.75L} \right)}}{{\left( {5.645L + a - 3.012b} \right)}} - 0.31} \right)}}{0.172}$$

#### Crust hardness

Crust hardness was measured based on the method described by Szczesniak (2002) at three equidistant points on the top of three toast bread loaves per batch using a texture analyser (TA-XT plus, Stable Micro Systems, England) and a cylindric aluminium probe with 4 mm diameter. The TA settings were: pre-speed 2 mm/s; test speed 1.7 mm/s; back speed 10 mm/s; target parameter path; path 5 mm; triggering force 0.098 N.

#### Concavity—divergence of optimal toast bread shape

Three toast bread loaves were cut into 13 mm slices using a bread slicer (B100, Bizerba GmbH & Co. KG., Germany), the slices were numbered and three slices (no. 4, 12, 20) were taken to determine concavity with a 2D pore structure analyser (C-Cell, Calibre Control International LTD, England). Concavity is defined as the area missing from the slice of bread compared to the maximal possible area if the edges were connected with a straight line. Three slices each from three different toast breads were analysed.

#### Crumb hardness

Three pairs of slices (no. 3 and 4, 11 and 12, 20 and 21) from the three toast bread loaves were analysed for crumb hardness. The above mentioned Texture Analyser with a 35 mm diameter cylindrical aluminium probe was set up according to the parameters defined by Szczesniak (2002): pre-speed 1.00 mm/s, test speed 5.0 mm/s, back speed 5.00 mm/s, target parameter path, path 15 mm, triggering force 0.049 N.

#### a_w_-Value of the crust

The crust of three toast bread loaves was grated and finely ground in a rotor beater mill (ZM 200, Retsch GmbH, Germany) at 12′000 rpm using a 0.5 mm sieve. The a_w_-value was measured using a water activity instrument (LabMaster-a_w_, Novasina AG, Switzerland) at 25 °C.

#### Data evaluation

A statistic software (RStudio, RStudio, Inc, USA) was used to apply Kruskal–Wallis-test and Wilcoxon-test (α = 0.05) to the data and to gain box plots. Significantly different results are marked via different letters in the graphs. Boxes of the box plots show interquartile range with the median as bold bar, whiskers indicate minimum and maximum values within lower and upper quartile, respectively and small dots mark values outside the interquartile range.

## Results and discussion

### Toast bread resilience and shape

**Fig. 2:**
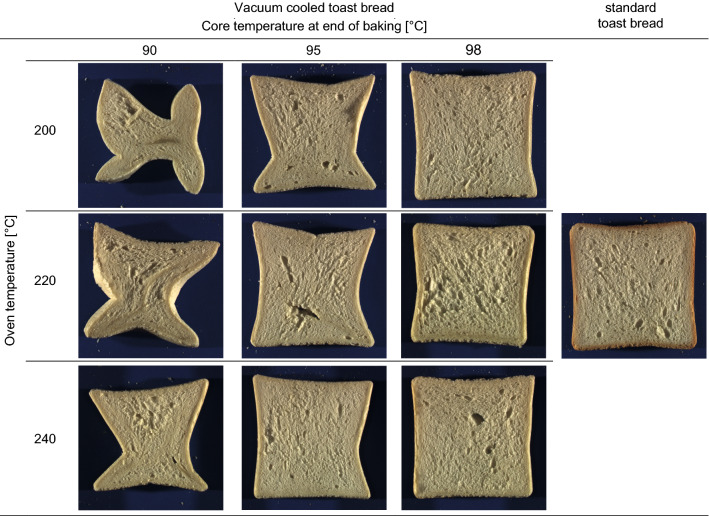
Nine representative toast bread loaves for all vacuum cooling trials (left) plus standard loaf (right). The core temperatures measured in the vacuum cooled loaves at the end of baking increases from left to right, oven temperature increases from top to bottom

Baking trials revealed that both oven and core temperature had an influence on the overall shape of the bread slices (Fig. 2). The lower the oven and core temperature, the more likely it was that the shape of the bread collapsed. Collapse sometimes occurred within minutes of completion of vacuum cooling, but in some cases took up to 1 h. This effect might be explained with observations made by Ben Aissa et al. (2010) and modelling performed from Zanoni et al. (1995). Both point out the clear influence of bread core temperature and exposure time to stabilise the crumb by ensuring a sufficient degree of gelatinisation. Thus, only a core temperature of 98 °C at the end of baking led to well-shaped toast bread loaves. In addition, the shape of the corners of the toast bread loaves differed considerably, depending on whether they had undergone standard or vacuum cooling. While standard cooling resulted in typical rounded corners, after vacuum cooling the loaves had sharp corners, similar to those of the tin. This is hypothesized to be a consequence of a volume maximisation in the tins upon the application of reduced pressure.

### Core temperature during vacuum cooling

**Fig. 3:**
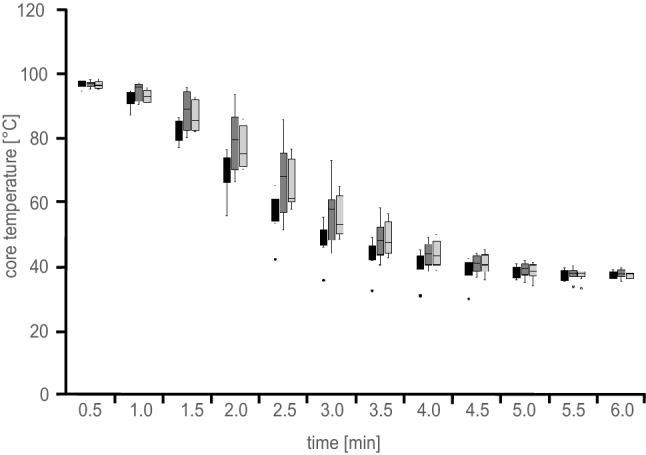
Core toast bread temperature during vacuum cooling depicted as mean values (*n* = 9) of toast bread loaves baked at 200 (light grey), 220 (dark grey) and 240 °C (black)

Vacuum cooling resulted in a sigmoidal core temperature curve with a typical initially steep decrease (Fig. 3) as also shown by Le-Bail et al. (2011). Changes in oven temperature or final core temperatures had no clear influence on the core temperature curve during the vacuum cooling process. This result corresponds with literature, where marginal differences in initial temperature were found to be of little relevance for the curve progression during vacuum cooling (McDonald and Sun, 2000). The results also show the speed of the temperature drop achieved through vacuum cooling, resulting in a cooling time of about 6 min. Grenier et al. (2002) investigated times necessary to achieve a core temperature of 25 °C applying conventional chilling in tin loafs and determined chilling times between 122 and 89.5 min at air temperatures ranging from 20 to 5 °C, respectively. Based on the graphical illustrations (Grenier et al. 2002), the cooling time required to achieve an end temperature of 40 °C was 60–70 min. Thus, vacuum cooling can provide a significant increase in productivity by cutting cooling times by a factor of approximately 10 in the present study.

### Weight loss during baking and vacuum cooling

**Fig. 4:**
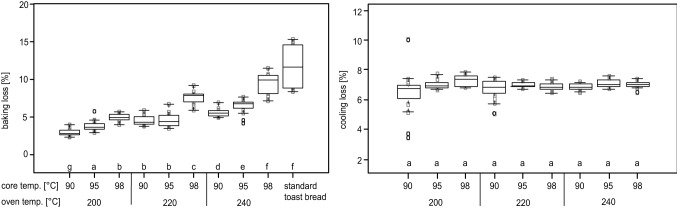
Weight loss during baking (left) and during vacuum cooling (right) for vacuum cooled toast bread loaves baked at different oven temperatures (200 °C / 220 °C / 240 °C) and end core temperatures after baking of 90 °C / 95 °C / 98 °C, followed by vacuum cooling to 35 °C compared to values for loaves baked using the standard process. Letters mark groups of significant differences between the samples (*p* =  < 0.05)

The loss in weight during baking ranges from approximately 4% for the lowest to approximately 12% for the highest oven and bread core temperatures (Fig. 4). These results are close to the standard value of 10% baking loss reported by Schiraldi and Fessas (2003) or Bredariol et al. (2019). Within trials at identical oven but different end core temperature, the oven temperatures 200 and 240 °C resulted in significantly different baking losses while for 220 °C baking losses were not significantly different for core temperatures of 90 and 95 °C. Baking losses at different oven temperatures (200, 220 and 240 °C) but identical end core temperature showed significantly different results irrespective for all tested end core temperatures. Baking loss of standard bread differed significantly from all other baking protocols with the exception of a baking at 240 °C to a core temperature of 98 °C. Still, a trend in weight loss is observable with higher weight loss attributable to higher oven and core temperatures. In fact, these findings are in line with thermal kinetics based expectations described by Perez-Nieto et al. (2010) and Ahrné et al. (2007) as well as Ureta et al. (2019) where a dependency of water loss on baking time and oven temperature was proven.

The loss of weight for all of the toast bread loaves during vacuum cooling was 6.91% ± 0.63%, showing that there were no significant differences, regardless of oven temperature or core bread temperature after baking (Fig. 4). Zhang et al. (2015) showed similar relationships in a heat and mass transfer modelling of vacuum cooling for porous food material where initial material temperature had little effect on the vacuum cooling process. Everington (1993) stated that a decrease of 10 °C during vacuum cooling results in an approximate 1% loss of mass. Since the ∆T for a drop in core bread temperature from 98 °C to 35 °C is 63 °C, the water loss of about 6.9% agrees well with the findings of Everington (1993) and McDonald and Sun (2000). In comparison, water loss for standard toast breads was about 1% in similar trials.

For vacuum cooled toast breads, the summed up water loss during baking and cooling is between approximately 10 and 17% (Fig. 4). The upper level of 17% (baked at 240 °C to 98 °C bread core temperature) is higher than the mean value of the water loss that occurs during the standard baking and cooling process. Consequently, as suggested also by several authors (Primo-Martin et al. 2008; McDonald and Sun 2000), with an adapted baking protocol, the total water loss can be reduced, e.g. 12% at 200 °C oven temperature and 98 °C bread core temperature resulting in good bread quality (Fig. 2). This shows the potential of the application of vacuum cooling through downsizing the entire production process.

### Specific volume and concavity

**Fig. 5:**
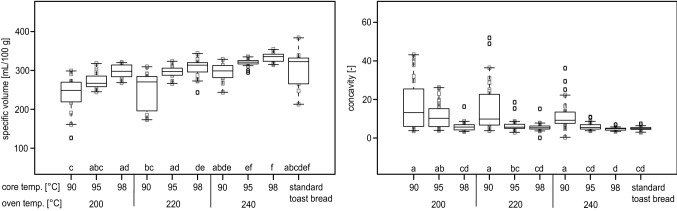
Specific volume (left) and concavity (right) of vacuum cooled toast bread loaves baked at different oven temperatures (200 °C / 220 °C / 240 °C) until defined core temperatures of either 90 °C, 95 °C or 98 °C were achieved, before being subsequently vacuum cooled to 35 °C, in comparison to standard toast bread. Letters mark groups of significant difference between the samples (*p* =  < 0.05)

Two effects were observable in terms of the specific volume of the toast breads. Firstly, the higher the oven and the core temperature was, the higher the resultant specific volume (Fig. 5) which was shown in a similar way without vacuum cooling by Villarino et al. (2014) or Bredariol et al. (2019). In addition, the lower both temperatures were, the higher the variance within samples produced at identical settings. Both effects reflect the observations described in Fig. 2 well where it was shown that toast bread loaves baked at lower temperatures, have a lower specific volume and a higher variance, tend to be less stable and, as a result, collapse over time. However, significant differences in specific volume were only observable for 90 °C compared to 95 and 98 °C bread core temperatures at oven temperatures of 200 and 220 °C whereas at 240 °C 90 and 98 °C bread core temperature led to significantly different results, only. Analysis of trends in concavity also supports this observation: concavity is the lowest when volume is high and when the overall shape of the toast bread is optimal (Fig. 5). However, most results on concavity do not differ significantly from each other and results are, hence, mere trends only.

There was no significant statistical difference between the standard toast bread and all of the vacuum cooled toast bread loaves in terms of specific volume which was also found by Le-Bail et al. (2011). The statistical variance of the values of specific volume of the standard toast bread was relatively high. In contrast, the variance in concavity for standard toast bread was one of the lowest of all samples. Thus, only toast breads baked to 90 °C bread core temperature at all three oven temperatures showed significant differences in concavity to standard toast bread. This finding confirms visual appearance shown in Fig. 2, where the lower the oven and bread core temperatures were, the higher the variance in concavity was. The observed relationships between processing parameters, concavity and specific volume are comparable to results from previous investigations into baking temperature, cooling temperature and cooling time for the standard baking process (Villarino et al. 2014; Ben Aissa et al. 2010). A possible explanation for the observed effects of oven and core temperatures on bread volume and concavity is provided by Ben Aissa et al. (2010) and Bosmans et al. (2013). The authors attribute structural support to the degree of starch gelatinization, which is directly dependent on baking temperature and time, and the resulting crumb firmness.

### Crust and crumb hardness

**Fig. 6:**
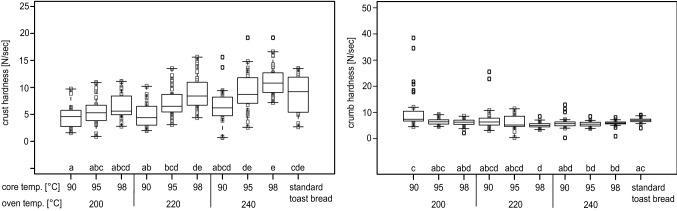
Crust hardness (left) and crumb hardness (right) of vacuum cooled toast bread baked at different oven temperatures (200 °C / 220 °C / 240 °C) until defined core temperatures (90 °C / 95 °C / 98 °C) were achieved, followed by vacuum cooling to 35 °C, in comparison to standard toast bread. Letters mark groups of significant difference between the samples (*p* =  < 0.05)

Both, higher oven and core temperatures showed only trends to increased crust hardness (Fig. 6) compared to Primo-Martin et al. (2008). The authors report that vacuum cooling led to significantly higher crust hardness due to vacuum cooling for unmolded bread rolls. However, their trials did not include baking time reduction. Standard toast bread exhibited a wide variance in crust hardness which did not significantly differ from most of vacuum cooled breads. Even less influence of oven and bread core temperature on crumb hardness (Fig. 6) was observed, so most of the vacuum cooled toast breads showed similar crumb hardness as standard toast bread. Bredariol et al. (2019) found a significant correlation between oven temperature and crumb hardness, however, it was for non-vacuum cooled and unmolded breads.

The absolute values for crust and crumb hardness were similar. The reason for this phenomenon is that force measurements are directly dependent on the geometry used for measurement: both crust and crumb hardness were measured with a cylindrical aluminium probe, however the diameter was 4 mm for crust hardness and 35 mm for crumb hardness measurements.

### a_w_-Value of the crust

The a_w_-values of the crust of the vacuum cooled toast bread loaves ranged from 0.43 to 0.63. Values decreased with increasing core temperatures and as oven temperature. Karaoglu (2006) reports the same coherence for non vacuum cooled rye bread. With a value of 0.53 the a_w_-value of the standard toast bread lay in-between the minimum and maximum a_w_-values of the vacuum cooled toast breads. This result is comparable to the a_w_-value of the vacuum cooled toast bread loaf of 0.49 which was baked at 200 °C oven temperature up to 98 °C bread core temperature and resembles regularly cooled toast bread best with respect to shape and water loss.

### Browning index

Oven temperature showed a slightly stronger influence on the browning index than final bread core temperature. The same positive correlation for oven temperature and lowered L values, which is the main colour indicator for browning, was reported by Pour-Damanab et al. (2014). Measured browning indices ranged between 16 for the toast bread loaves with least thermal load (baked at 200 °C up to 90 °C bread core temperature) and 55 for the one with the highest thermal load (baked at 240 °C up to 98 °C). There was an optical and significant difference, between the standard toast bread with a browning index of 55 and all vacuum cooled toast breads except the one baked at 240 °C up to 98 °C bread core temperature. This limited browing could be a further advantage of vacuum cooling as a growing number of consumers is in favour of crustless toast bread.

## Conclusion

It was shown that cooling time can be significantly reduced through the application of vacuum cooling and that the quality of toast bread produced by the vacuum cooling protocol matched standard toast bread satisfactorily. The experimental setup presented in this research achieved a tenfold reduction in cooling time, which will save both time and space in a bakery. However, the results also reveal that an adaptation to the baking protocol is necessary in order to achieve optimal toast bread quality when applying vacuum cooling. Toast bread stability and weight loss were strongly influenced by the baking protocol and a tendency for improved crust hardness and concavity were also observed. The results indicate that a minimum core temperature at the end of baking of 98 °C is necessary if the breads are to retain their shape during and after vacuum cooling. However, no significant effect in terms of weight loss during cooling was observed as a result of the baking protocol and crumb hardness was comparable for all of the tested toast bread loaves.

Further work is needed to test additional systematic variations to the baking and vacuum cooling protocols and special attention paid to the pressure curves during vacuum cooling to further improve time savings and product quality.

## References

[CR1] Ahrné L, Andersson CG, Floberg P, Rosén J, Lingnert H (2007). Effect of crust temperature and water content on acrylamide formation during baking of white bread: Steam and falling temperature baking. LWT-Food Sci Technol.

[CR2] Ben Aissa MF, Monteau JY, Roelens G (2010). Volume change of bread and bread crumb during cooling, chilling and freezing, and the impact of baking. J Cereal Sci.

[CR3] Besbes E, Jury V, Monteau J-Y, Le Bail A (2013). Characterizing the cellular structure of bread crumb and crust as affected by heating rate using X-ray microtomography. J Food Eng.

[CR4] Bosmans G, Lagrain B, Fierens E, Delcour J (2013). The impact of baking time and bread storage temperture on bread crumb properties. Food Chem.

[CR5] Bredariol P, Spatti M, Vanin FM (2019). Different baking conditions may produce breads with similar physical qualities but unique starch gelatinization behaviour. Food Sci Technol.

[CR6] Everington DW (1993) Vacuum technology for food processing. Food Technology International Europe, pp 71–74

[CR7] Grenier A, Monteau J-Y, Le Bail A, Hayert M (2002). Effect of external conditions on the rate of post-baking chilling of bread. J Food Eng.

[CR8] Karaoglu MM (2006). Effect of initial baking and storage time on pasting properties and aging of Par-baked and rebaked rye bread. Int J Food Properties.

[CR9] Le-Bail A, Leray G, Perronnet A, Roelens G (2011). Impact of the chilling conditions on the kinetics of staling of bread. J Cereal Sci.

[CR10] Li LL, Zhang M, Adhikari B, Gao ZX (2017). Recent advances in pressure modification-based preservation technologies applied to fresh fruits and vegetables. Food Rev Int.

[CR11] McDonald K, Sun DW (2000). Vacuum cooling technology for the food processing industry: a review. J Food Eng.

[CR12] Mc Donald K, Sun DW (2001). Pore size distribution and structure of a cooked beef product as affected by vacuum cooling. J Food Process Eng.

[CR13] Novotni D, Spoljaric IV, Drakula S, Cukelj N, Voucko B, Scetar M, Galic K, Curic D (2017). Influence of barley Sourdough and vacuum cooling on shelf life quality of partially baked bread. Food Technol Biotechnol.

[CR14] Palou E, Lopez-Malo A, Barbosa-Canovas GV, Welti-Chanes J, Swanson BG (1999). Polyphenoloxidase activity and color of blanched and high hydrostatic pressure treated banana puree. J Food Sci.

[CR15] Perez-Nieto A, Chanona-Perez J, Farrera-Rebollo R, Gutierrez-Lopez G, Alamilla-Beltran L, Calderon-Dominguez G (2010). Image analysis of structural changes in dough during baking. Food Sci Technol.

[CR16] Primo-Martin C, de Beukelaer H, Hamer RJ, van Vliet T (2008). Fracture behaviour of bread crust: effect of bread cooling conditions. J Food Eng.

[CR17] Pour-Damanab AS, Jafary A, Rafiee Sh (2014). Kinetics of the crust thickness development of bread during baking. J Food Sci Technol.

[CR18] Rodrigues LGG, Cavalheiro D, Schmidt FC, Laurindo JB (2012). Integration of cooking and vacuum cooling of carrots in a same vessel. Ciencia E Tecnol De Alimentos.

[CR19] Rodrigues LGG, Cavalheiro D, Schmidt FC, Laurindo JB (2013). Possibilities for integrating cooking and vacuum cooling of potatoes in the same vessel. J Food Process Preservation.

[CR20] Roth K (2007). Unser tägliches Brot. Chemie unserer Zeit.

[CR21] Schiraldi A, Fessas D (2003) The role of water in dough formation and bread quality. In: Cauvain SP (ed) Bread making. Woodhead Publishing Limited, Cambridge, ISBN 1 85573 553 9

[CR22] Sun DW, Zheng L (2006). Vacuum cooling technology for the agri-food industry: past, present and future. J Food Eng.

[CR23] Sun DW, Wang L (2000). Heat transfer characteristics of cooked meats using different cooling methods. Int J Refrigeration.

[CR24] Szczesniak AS (2002). Texture is a sensory property. Food QualPreference.

[CR25] Ureta MM, Diascorn Y, Cambert M, Flick D, Salvadori VO, Lucas T (2019). Water transport during bread baking: Impact of the baking temperature and the baking time. Food Sci Technol Int.

[CR26] Villarino CB, Jayasena V, Coorey R, Chakrabarti-Bell S, Johnson S (2014). The effects of bread-making process factors on Australian sweet lupin-wheat bread quality characteristics. Int J Food Sci Technol.

[CR27] Zanoni B, Peri C, Bruno D (1995). Modelling of starch gelatinization kinetics of bread crumb during baking. Lebensmittelwissenschaften Technol.

[CR28] Zhang Z, Gao J, Zhang S, Xie Y, Zhao L (2015). Heat and mass transfer modeling of vacuum cooling for porous food material. Bulgarian Chem Commun.

[CR29] Zheng LY, Sun DW (2004). Vacuum cooling for the food industry—a review of recent research advances. Trends Food Sci Technol.

[CR30] Zhu Z, Li Y, Sun D-W, Wang H-W (2019). Developments of mathematical models for simulating vacuum cooling processes for food products—a review. Critic Rev Food Sci Nutr.

